# Supplementary material for: Comparing the efficiency of paper-based and electronic data capture during face-to-face interviews
Source: PLoS One. 2021 Mar 8;16(3):e0247570. doi: 10.1371/journal.pone.0247570 (PMC7939350; doi:10.1371/journal.pone.0247570)
Supplement: Attachment — Submitted filename: Response to reviewer_Final 2.docx [file pone.0247570.s001.docx]

PLOS One

Dear Dr Moutopoulos,

Please consider our revised manuscript entitled: ‘Comparing the accuracy, practicality and cost-effectiveness of paper-based and electronic data capture during face-to-face interviews (Manuscript ID PONE-D-20-28545).

We express our appreciation to the reviewer and editor for their interest and time spent reviewing the manuscript. We have carefully considered all these comments, particularly statements about the term ‘error rate’ and the potential confusion around its meaning. We agree with this completely and have adjusted the manuscript accordingly to provide the reader with more clarity.

We have now removed the funding statement from the Acknowledgements section and would like to edit the Funding statement to now read “Funding was provided by the Department of Primary Industries and Regional Development”.

We now have permission from the original copyright holder of Figure 1 to publish the content specifically under the CC BY 4.0 license. The completed Content Permission is attached to this submission and the caption for Figure 1 now included the appropriate text.

Thank you again for consideration of our revised manuscript. We appreciate your time and look forward to your response.

Yours faithfully,

Alissa Tate

Email: alissa.tate@dpird.wa.gov.au

11 01 2021

| **#** | **Comment** | **Response** | **Line** |
| --- | --- | --- | --- |
| **Editor comments** | | | |
| 1 | The authors should clarify the terminology of certain aspects of errors (e.g. accuracy, efficiency). For consistency reasons error rate’ may confuse the reader in suggesting it only refers to errors and not missing data. | We thank the reviewer for this suggestion. The term ‘Error rate’ has now been changed throughout the document to ‘Inaccuracy rate’ which will provide more clarity to readers. | 23, 31, 72, 77, 166, 167, 173, 208, 220, 222, 226, 227, 307 |
| 2 | Title should be also reconsidered. | Agreed. The title has been now been changed to: ‘Comparing the accuracy, practicality and cost-effectiveness of paper-based and electronic data capture during face-to-face interviews. | 1 |
| 3 | In the results, authors should clarify the sampling strategy. | We thank the editor for their comment. The sampling strategy has been described in the methods and supported by additional peer-reviewed papers. However, we have also added to the results to provide more clarity about the sampling strategy applied in this study. | 197 |
| 4 | Also, which are the geographical constraints that should be taken into account to make the case study expand in broader spatial magnitude to be a controllable and well-defined example? | There are various places in the discussion where we have highlighted the challenges and requirements for this work. We thank the reviewer for this comment and have edited the relevant paragraph in the discussion to now read  *“In this case study it was only necessary to use a single tablet device as only one survey was occurring at a time. However, the spatial and temporal scales of a project are important to consider when replicating this study using EDC, as multiple, concurrent devices may be required which has implications in terms of the cost of equipment, software capability and ability to trouble-shoot issues arising in remote locations.”* | 371 |
| 5 | A table is missing from the submitted manuscript (in line 222 Table 5 was wrongly mentioned instead of Table 4) | We thank the editor for their feedback. This table was removed prior to submission and the cross-reference has now also been removed in the text. | 255 |
| 6 | The authors should be consistent with the journal guidelines especially in the cross-reference with the figure (e.g. Fig. 1, Fig 3, Figure 2) | Agreed. All cross-reference have now been checked and changes made. | 101, 107, 205 |
| 7 | Table 3 it might be useful for the audience to have a clear picture of the results by showing totals for error and missing data (ie., combining both interviewer and scribe). | Agreed. Additional rows have been added to Table 3 which include the totals for both error and missing data. | 259 |
| **Reviewer comments** | | | |
| 8 | Please ensure that you refer to Figures 2 and 3 in your text as, if accepted, production will need this reference to link the reader to the figure. | Agreed. All cross-reference have now been checked and changes made. | 205 , 215, 229 |
| 9 | Please include a copy of Table 5 which you refer to in your text on page 12. | We thank the reviewer for their feedback. This table was removed prior to submission and the cross-reference has now also been removed. | 255 |
| 10 | Title (lines 1 and 2): If the character limit allows, I would suggest the Title lists exactly what the study investigated: accuracy, practicality and cost-effectiveness. ‘Efficiency’ is somewhat of a vague term in my opinion. | Agreed. The title has now been changed to read: ‘Comparing the accuracy, practicality and cost-effectiveness of paper-based and electronic data capture during face-to-face interviews. | 1 |
| 11 | Abstract (lines 18, 19): Consider including here exactly what the direct comparison was about: accuracy, practicality, and cost-effectiveness | Agreed. The relevant lines have been changed to read:  “A roving creel survey of recreational shore-based fishers in Western Australia in 2019 enabled a direct comparison of the accuracy, practically and cost-effectiveness between the two methods.” | 20 |
| 12 | Abstract (line 21); ‘Error rate’: Here and elsewhere in the paper (e.g., keywords, lines 188, 201, 203, etc), whenever the authors are referring to the total inaccuracies (missing data and errors) should use the term ‘inaccuracy rate’ rather than ‘Error rate’. ‘Error rate’ may confuse the reader in suggesting it only refers to errors, and not missing data. | We thank the reviewer for this suggestion. The term ‘Error rate’ has now been changed to ‘Inaccuracy rate’ throughout the document to provide more clarity to the reader. | 23, 31, 72, 77, 166, 167, 173, 208, 220, 222, 226, 227, 307 |
| 13 | Introduction (line 68): For consistency with the terminology used in other parts of the paper, authors should use the term ‘practicality’ rather than ‘timeliness’ | Agreed. The term ‘Timeliness’ has now been changed to ‘Practicality’ throughout the document. | 73 |
| 14 | Methods, Study area (line 93): This seems to refer to figure 1, but there is an error in the cross-reference to this Fig. | Agreed. The cross-reference has been changed to Fig. 1 | 101 |
| 15 | Table 1- The authors should consider replacing the term “errors” by “inaccuracies” in both the table caption and header. As noted above, this may confuse the reader as error is one of the two types of inaccuracies analyzed by the authors. | Agreed. To remain consistent throughout the document we have changed the term ‘errors’ to ‘inaccuracies’ | 165 |
| 16 | Results (line 184): This seems to refer to figure 2, but there is an error in the cross-reference to this Fig. Note also that the Figure captions seem to be missing from the submitted PDF. | Agreed. The cross-reference has been changed to Fig. 2 | 197 |
| 17 | Results (line 189): Could the authors clarify if each survey day had the exact same number of interviews. If so, than the average number of inaccuracies per day (as noted above, ‘inaccuracies’ seems a better term to use here, as this seems to refer to both missing data and error in data entered) seems appropriate. If not (ie., if each survey day had different number of completed interviews), than the average number of inaccuracies per filled interview within each month would seem to be a more appropriate metric to use. | We thank the reviewer for raising this point. The number of completed interviews is different across each survey day.    Due to how inaccuracies from various sections of the interview were recorded during the study, we are unable to calculate a mean number of inaccuracies per interview. The number of inaccuracies could be affected by the number of survey locations visited in each month and the number of interviews completed in each month. Due to the nature of the survey design, the number of locations visited was consistent across each month and would have limited effect on the number of inaccuracies. The number of interviews could be affected by other factors such as environmental condition, however, still only showed limited variability between months (224 – 320 interviews). We have added additional information into the results regarding the number of locations surveyed and number of interviews completed within each month to better understand the relationship between these values and the number of inaccuracies. We have reworded the results text accordingly to reflect these changes and figure 2 has also been updated. | 202 - 210 |
| 18 | Table 3 – the authors should consider adding totals for error and missing data (ie., combining both interviewer and scribe) to Table 3. This would help the reader in interpreting this result. | Agreed. Additional rows have been added to Table 3 which include the totals for both errors and missing data. | 250 |
| 19 | Results (line 222): There is reference to Table 5 in the text, but no reference to Table 4, and both Tables 4 and 5 seem to be missing from the submitted PDF. | Agreed. As above both tables were removed prior to submission and the cross-reference for both table 4 and 5 have now been removed from the document | 257 |
| 20 | Discussion (line 266): This percentage of 0.7% seems to be mentioned for the first time. The authors should consider including it somewhere in the Results, so the reader clearly understands where this is coming from. | We thank the reviewer for this comment. The percentage refers to the additional data entry required in PDC. The reported value was incorrect and should have read 0.9%, this has now been changed and Table 3 cross-referenced to provide more clarity | 313 |
|  | Discussion (line 275): based on the information provided in the Methods, all of the questions or fields included in the survey seemed to be multiple option, or for relative few characters (if a free text field). It would be potentially interesting if the authors could add a note or two on the potential practically of EDC vs PDC in the use of open-ended questions with potentially long responses (e.g., opinion on existing regulations). | The majority of questions included in this survey were indeed multiple options or short free text field (i.e., entering total length or number of fishers). We agree with the reviewer that the practicality of EDC could change if question were open-ended and opinion-based. We have added a sentence to further discuss the flexibility of PDC for surveys which have open-ended questions, but to also identify the developments in technology around voice-to-text or digital scribing tools may solve this issue for EDC.  *‘For studies where open-ended and opinion based questions are asked, the flexibility of PDC may be more efficient at this time, however, developments in technology around voice-to-text or digital scribeing tools may solve this issue in the future.’* | 325 - 330 |
